# Treatment of Nocardial Brain Abscess in a Patient With Systemic Lupus Erythematosus and Idiopathic Thrombocytopenic Purpura: Case Report and a Review of the Literature

**DOI:** 10.7759/cureus.17498

**Published:** 2021-08-27

**Authors:** Lauren Harris, Ioana Raducanu, Hu Liang Low

**Affiliations:** 1 Neurosurgery, Queen’s Hospital, London, GBR; 2 Microbiology, Queen’s Hospital, London, GBR

**Keywords:** brain abscess, nocardia species, abscess wall, treatment, neurosurgery

## Abstract

Brain abscesses due to *Nocardia *species account for 1-2% of all cerebral abscesses, often in immunosuppressed individuals, with a mortality three times higher than other cerebral abscesses. Early diagnosis and management are vital for good outcomes.

We report a case of a right frontal *Nocardia *brain abscess in an immunosuppressed 38-year-old female. She presented with headaches, confusion, memory deficits, and personality change. She remained systemically well, with normal inflammatory markers. She underwent two open surgical drainages, with excision of the abscess wall. She made an excellent recovery with minimal edema and no contrast enhancement on imaging at eight weeks postoperatively.

Management of *Nocardia *brain abscess includes a prompt diagnosis with direct microscopic examination and initiation of correct antibiotic therapy for good outcomes. We recommend open surgical resection, including excision of the abscess wall, followed by long-term antimicrobial therapy, to enhance the rate of recovery.

## Introduction

Over 30 *Nocardia* species are associated with human diseases, of which *Nocardia asteroides*, *Nocardia brasiliensis*, *Nocardia farcinica*, and *Nocardia nova* are the most prevalent [1]. They are not a part of human saprophyte flora and are rarely contaminants [1]. The microorganism can be inhaled and spread hematogenously from the lungs to the brain or maybe inoculated directly through the skin. Nocardial infections usually present as isolated pulmonary infections, followed by disseminated disease. Nocardial brain abscesses are rare and almost always occur in the context of disseminated disease [2].

We report a very rare case of an isolated nocardial brain abscess in a patient with systemic lupus erythematosus (SLE) and idiopathic thrombocytopenic purpura (ITP). The patient was initially treated with aspiration of the abscess but it recurred. Open drainage and excision of the abscess wall were then carried out with excellent results. Here, we also present a review of the surgical treatment of nocardial brain abscesses.

## Case presentation

A 38-year-old, African American, right-handed female presented with three weeks of right-sided headache, confusion, memory deficit, and personality change. Her physical and neurological examination was otherwise unremarkable. Hematological, biochemical, and inflammatory markers were within the normal range.

Her medical history included treated non-Hodgkin lymphoma, asthma, hypertension, and bilateral cerebellar infarcts. Three months before presentation, she was diagnosed with SLE and ITP, for which she was commenced on prednisolone and rituximab, a B-lymphocyte depleting anti-CD20 monoclonal antibody. She denied any history of smoking, drinking, or the use of illicit substances.

**Figure 1:**
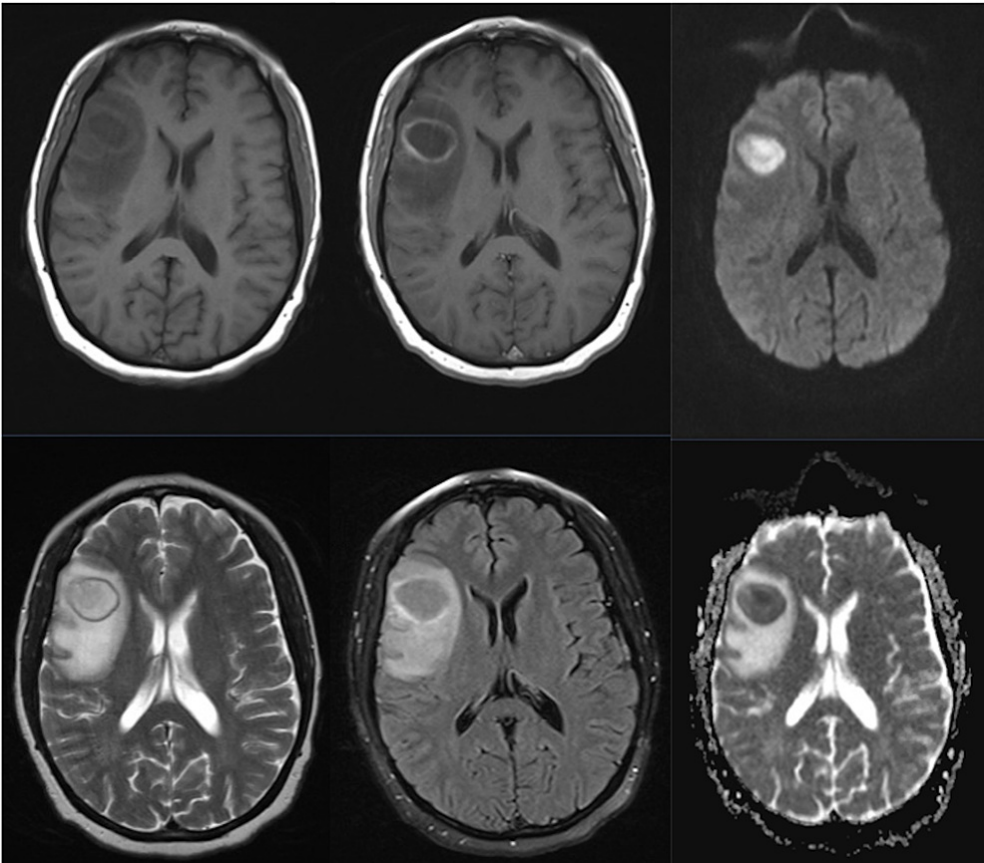
Preoperative MRI scan. Preoperative right frontal multiloculated, ring-enhancing lesion, with surrounding edema and restricted diffusion. First row: Preoperative T1- weighted, T1 + contrast, diffusion imaging. Second row: Preoperative T2-weighted, FLAIR, and ADC. ADC: apparent diffusion coefficient; FLAIR: fluid-attenuated inversion recovery; MRI: magnetic resonance imaging

Magnetic resonance imaging (MRI) revealed a multiloculated, ring-enhancing lesion within the right frontal pars triangularis (Figure 1). Enhanced computed tomography scans of her chest, abdomen, and pelvis revealed no abnormalities. Given her normal C-reactive protein levels and the lack of any extracerebral lesions, the favored diagnosis was a primary brain neoplasm. She underwent a neuro-navigated aspiration of the lesion and, to our surprise, thick pus was noted.

**Figure 2:**
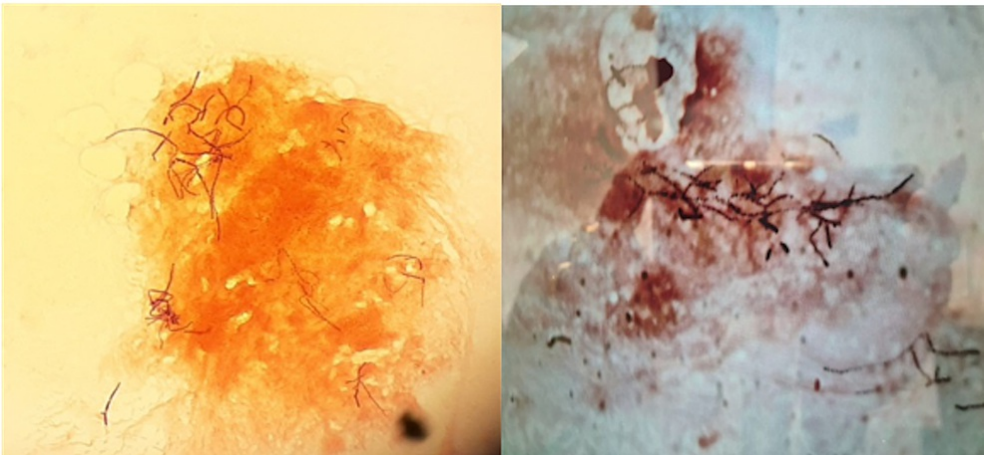
Nocardia isolate. Extensively branched gram-positive hyphae fragmented into coccoid elements (magnification 100×).

Examination of the pus and a portion of the cavity wall revealed pleomorphic gram-positive hyphae with extensive branching filaments (Figure 2). No neoplastic features were seen. The hyphae were then identified with a 99.9% certainty as *Nocardia farcinica* using matrix-assisted laser desorption/ionization-time-of-flight spectrometry and partial sequencing of 16S rDNA. A 100% certainty was not possible due to a 99% sequence homology between *Nocardia farcinica* and *Nocardia kroppenstedtii* [3]. Tests for acid-fast bacilli, human immunodeficiency virus, hepatitis B, and toxoplasma were all negative.

**Figure 3:**
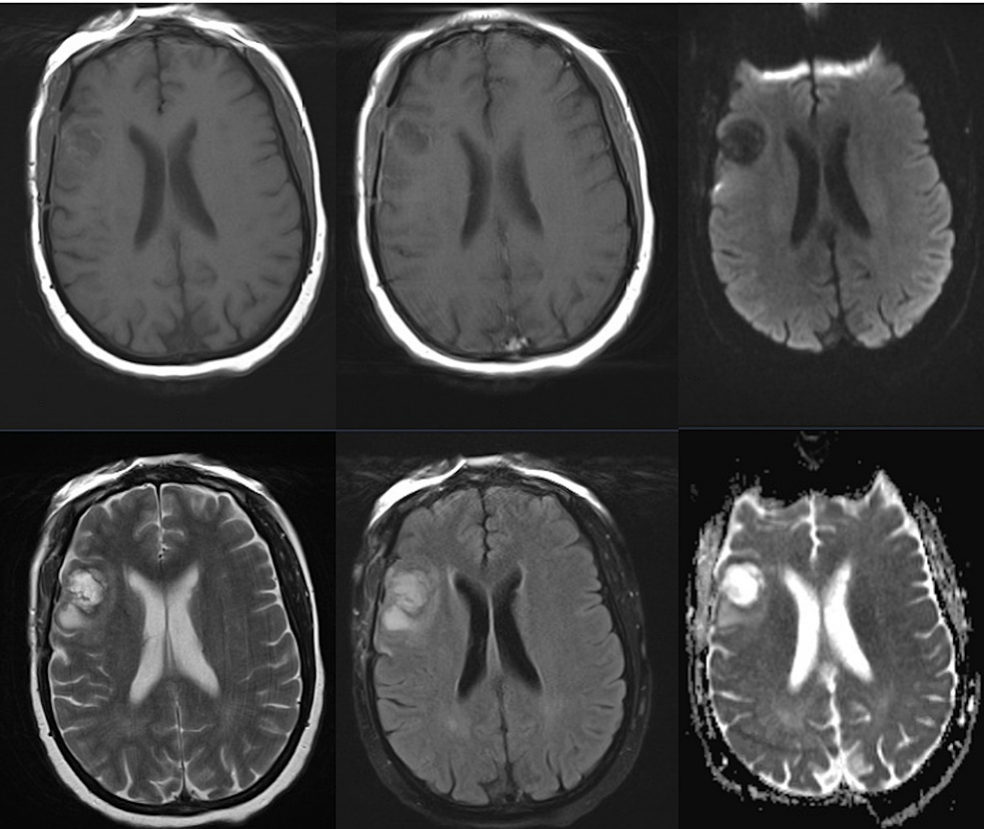
Two months postoperative MRI scan. Two months postoperative loss of enhancement of the residual abscess and minimal edema. First row: Two months postoperative T1-weighted, T1 + contrast, diffusion imaging. Second row: Two months postoperative T2-weighted, FLAIR, and ADC ADC: apparent diffusion coefficient; FLAIR: fluid-attenuated inversion recovery; MRI: magnetic resonance imaging

She was placed on a six-week course of intravenous meropenem 2 g three times a day, followed by 12 months of oral co-trimoxazole 2,400 mg four times a day. The immediate postoperative period was uncomplicated, and early and postoperative scans showed good decompression of the abscess. However, repeat scans three weeks later showed a significant recurrence of the lesion. At this time, she underwent open drainage of the abscess as well as the removal of the abscess walls. She responded very well to surgery, and repeat scans showed no recollection, along with complete removal of enhancing abscess walls at two months postoperatively (Figure 3).

To date, at the nine-month follow-up, the patient remains well, with no clinical or radiological evidence of recurrence. While the microbiology plan was for a one-year course of oral antibiotics, the patient was non-compliant and stopped at two months. MRI imaging was performed at two, four, and six months postoperatively, with continued improvement, minimal cavity, and no enhancement or perilesional edema. Clinically, she had two episodes of focal motor seizures at four months postoperatively, which resolved with 250 mg levetiracetam twice daily as advised by the neurology team. The initial neuropsychiatric symptoms resolved, and she resumed work after two months.

## Discussion

*Nocardia* species are aerobic gram-positive, catalase-positive, acid-fast bacteria, which produce branched, aerial hyphae up to 2 μm in diameter that often fragment into coccobacilli-shaped elements [1,4]. The diagnosis is challenging due to the non-specific presentation, the requirement for invasive diagnostic procedures, and the difficulty in the isolation and identification of the *Nocardia* species [3]. There are no serodiagnostic tests, and 16SrRNA may not definitively distinguish between different species of *Nocardia*, which exhibit different antibiotic susceptibilities [5-7]. Microscopic examination of Gram-stained specimens can give a rapid diagnosis as the thin gram-positive beaded branching filaments are pathognomonic [8].

*Nocardia* species are opportunistic pathogens that affect mainly immunosuppressed individuals. Steroid use appears to be an independent risk factor [9]. Our patient had been on steroids and rituximab prior to her infection.

As mortality from *Nocardia* is three times higher than that of other brain abscesses (30% versus 10%), early diagnosis and management are vital [10]. Good outcomes are expected with prompt diagnosis and initiation of appropriate treatment. Differentials include intrinsic or metastatic brain tumors, toxoplasmosis, meningioma, cerebral tuberculosis, and cerebral *Mycobacterium* non-tuberculosis infections [11-15]. Management options include surgery, antibiotics if the organism has been identified from a specimen, cerebrospinal fluid or blood cultures, or a combination [16,17]. Pathogen identification and antibiotic susceptibility tests are performed on the specimen.

In 1928, Sargent was the first to report a successful excision of a brain abscess [18]. Indications for surgery include reducing intracranial pressure, confirming the diagnosis, obtaining pus for microbiological diagnosis, enhancing the efficacy of antimicrobial therapy (particularly in fungal abscesses due to poor blood-brain barrier penetration of antifungals), and avoiding spread into the ventricles [18]. This must be balanced against the risks of surgery, particularly in eloquent or deep locations, and the patients’ comorbidities. Surgical options include image-guided stereotactic aspiration versus the more invasive craniotomy and excision, including the abscess wall where possible. Since the advent of stereotaxy and the development of better antibiotics, the majority of cerebral abscesses have been treated successfully with pus aspiration and long-term intravenous antibiotics.

Aspiration is associated with lower risks (mortality of 6.6% versus 12.7% for open drainage of brain abscesses) and is the treatment of choice for deep-seated lesions [19]. However, a case for open drainage of abscesses and partial or complete removal of the abscess wall can be made for superficial lesions, which involve the non-eloquent brain, are well-encapsulated, multiloculated, or contain foreign material. This is particularly pertinent in the case of nocardial abscesses as the lesions formed are often multiloculated and contain viscid pus that is not easily aspirated. Viable nocardial hyphae may also be found within the abscess walls, as demonstrated in our case. These factors may explain the high recurrence rate of nocardial abscesses [10]. Although there are no controlled, prospective studies comparing the outcomes of abscess aspiration to excision or its effect on the duration of antibiotic therapy, retrospective studies suggest that abscess excision is associated with a faster resolution of neurological deficits, low abscess recurrence, and a shorter duration of antibiotics treatment [20-26]. This must be balanced against the risks of a more invasive procedure, with the potential to damage the surrounding cortex and its associated function. In nocardial abscesses, the mortality rate has been documented as 24% after initial craniotomy and excision, 50% after aspiration and drainage, and 30% after non-operative therapy [10]. In our case, aspiration of the abscess was trialed first, with a rapid recurrence. We believe that the removal of the abscess wall during the second operation hastened the clinical recovery and contributed to the good long-term outcome in our patient.

*Nocardia* has been increasingly recognized in SLE, most commonly with manifestations in the lungs (81%), with central nervous system (CNS) involvement in 13% [27]. The presentation can be with seizures, headaches, focal neurological deficit, or can mimic an SLE flare-up [28]. *Nocardia* in SLE has a high mortality of 35%, increasing to 75% when the CNS is involved [27]. This mortality is related to a delay in diagnosis and the immunosuppressed state of the patient. A summary of *Nocardia* brain abscesses associated with SLE and ITP is presented in Table 1. To our knowledge, this is the second case of a *Nocardia farcinica *brain abscess in a patient with SLE. Of the 14 previously published studies, only one advocated excision of the abscess, with good clinical outcomes after six weeks of antibiotics [29].

**Table 1:** A summary of Nocardia species brain abscess in patients with systemic lupus erythematosus and idiopathic thrombocytopenic purpura (designated by *). F: female; M: male; TMP/SMX: trimethoprim-sulfamethoxazole (co-trimoxazole); MRSA: methicillin-resistant *Staphylococcus aureus*; MI: myocardial infarction

Author; Year	Age, Gender	Organism	Location	Presentation	Management	Duration	Outcome
Stewart and Basten; 1975 [30]	31, M	-	Parietal	Hydrocephalus	Aspiration	7 months	Died (MRSA sepsis)
Mc-Nab et al.; 2000 [31]	24, F	N. asteroides	Occipital	-	Cotrimoxazole, cefixime, shunting	-	-
Moitra et al.; 2003 [32]	-	N. asteroides	-	Headache	Biopsy, linezolid	-	-
Yoneyama et al.; 2004 [29]	2 patients, F	-	-	-	Excision, TMP/SMX, minocycline, cefotaxime	6 weeks	Good
Kilincer et al.; 2005 [33]	39, F	N. farcinica	Temporoparietal	Seizure	Aspiration, ceftriaxone, TMP/SMX, minocycline	12 months	Good
Cheng et al.; 2005 [28]	42, F	N. asteroides	Frontal	Headache	Stereotactic aspiration, TMP/SMX	12 months	Good
Justiniano et al.; 2007 [34]	37, M	N. asteroides	Multiple: frontal and occipital	Seizure, slurred speech, hemiparesis	Biopsy, TMP/SMX + linezolid	-	Good
Bashir and Ranganathan; 2010 [35]	54, F	N. asteroides	Multiple: frontal, temporal, cerebellar	Headache, confusion	TMP/SMX	12 months	Good
Ueda et al.; 2014 [36]	69, M	N. elegans	Cerebellar	-	TMP/SMX and clarithromycin	6 months	Good
Jeong et al.; 2017 [37]	51, M	N. asiatica	Multiple: supratentorial and cerebellar	Hemiparesis, dysarthria, dizziness, vomiting, fever	Aspiration, TMP/SMX, and ceftriaxone	10 months	Died
*Barata et al.; 2000 [38]	40, F	*Nocardia *(not specified)	Frontal	Headache, vomiting	Aspiration, TMP/SMX	10 months	Good
*Wakhlu et al.; 2004 [39]	42, M	N. asteroides	Frontal	Hemiparesis	Aspiration, ceftriaxone, amikacin, TMP/SMX	12 months	Good
*Lai et al.; 2005 [40]	68, M	N. farcinica	Parietal, temporal	Headache	TMP/SMX	12 months	Good
*Frank et al.; 2010 [41]	83, M	*Nocardia *(not specified)	Cerebellar, occipital	Dysarthria, ptosis, tremor, ataxia	Meropenem, TMP/SMX	2 months	Died (MI)
Present case	38, F	N. farcinica	Frontal	Headache, personality change	Excision, meropenem, TMP/SMX	12 months	Good

## Conclusions

Nocardial abscesses should be included in the differential in immunocompromised patients presenting with brain lesions. As there are no serological or hematological markers of nocardial infections in the brain, and the mortality is high, early acquisition and examination of material from the lesion either via biopsy or open surgery are essential. Open surgical drainage with excision of the abscess walls followed by long-term antimicrobial therapy may be more effective than the simple aspiration of nocardial abscesses.
